# A case of refractory systemic lupus erythematosus with monocytosis exhibiting somatic KRAS mutation

**DOI:** 10.1186/s41232-022-00195-w

**Published:** 2022-04-01

**Authors:** Sze-Ming Law, Shuji Akizuki, Akio Morinobu, Koichiro Ohmura

**Affiliations:** 1 Department of Rheumatology and Clinical Immunology, Graduate School of Medicine, Kyoto University, 54 kawahara-cho, Shogoin, Sakyo-ku, Kyoto, 606-8507 Japan; 2 Department of Rheumatology, Kobe City Medical Center General Hospital, Kobe, Japan

**Keywords:** Systemic lupus erythematosus, Somatic mutation, Monocytosis, KRAS

## Abstract

**Background:**

Systemic lupus erythematosus (SLE), an autoimmune disorder that damages various organ systems, is caused by a combination of genetic and environmental factors. Although germline mutations of several genes are known to cause juvenile SLE, most of the susceptibility genetic variants of adult SLE are common variants of the population, somatic mutations that cause or exacerbate SLE have not been reported. We hereby report a refractory SLE case with monocytosis accompanying somatic KRAS mutation that have been shown to cause lupus-like symptoms.

**Case presentation:**

A 60-year-old female patient who had been diagnosed with SLE was admitted to our hospital. Although prednisolone and tacrolimus treatments had kept her thrombocytopenia and anti-DNA Ab level at bay for more than 4 years, a diagnosis of transverse myelitis was made when she became acutely ill with pleocytosis. Elevated cells (predominately monocytes), protein, IgG, and IL-6 levels were also found in the cerebrospinal fluid (CSF) of the patient. Standard pulse treatments of methylprednisolone, high-dose of prednisolone, and intravenous cyclophosphamide in combination with plasma exchange could not alleviate the refractory neural and autoimmune manifestation. Monocytosis of peripheral blood was also noted. Flow cytometric analysis revealed elevated ratio of CD14+CD16+ atypical monocytes, which excluded the possibility of chronic myelomonocytic leukemia. Lupus-like symptoms with monocytosis reminded us of Ras-associated autoimmune leukoproliferative disorder, and Sanger sequencing of KRAS and NRAS genes from the patients’ peripheral blood mononuclear cells (PBMC), sorted CD3+ lymphocytes and CD14+ monocytes, and cerebrospinal fluid were performed. An activating KRAS somatic mutation was found in the patients’ DNA at the time of encephalomyelitis diagnosis.

**Conclusion:**

Somatic mutations of some genes including KRAS may cause the refractoriness of SLE.

## Background

Systemic lupus erythematosus (SLE) is a chronic autoimmune disease that affects any parts of the body, characterized by the production of autoantibodies. Cumulative evidences have pointed to the conglomerate of genetic and environmental factors that constitute the pathogenesis of SLE.

Although SLE is a polygenic disorder, monogenic cases have been reported [1]. Such monogenic SLE patients can be grouped into interferonopathy-like, self-tolerance breakdown-associated, complement deficiency-like, RASopathy-like, and others. RASopathies are rare neurodevelopmental syndromes that are caused by dominantly inherited mutations in several genes within the RAS/MAPK pathway (KRAS, NRAS, PTPN11, RAF, SHOC2, and SOS1). Meanwhile, Ras-associated autoimmune leukoproliferative disorder (RALD) is caused by somatic, gain-of-function NRAS or KRAS mutation within hematopoietic lineage. It was initially reported in a patient presenting autoimmune lymphoproliferative syndrome (ALPS)-like symptom with normal FAS-mediated apoptotic pathway [2]. Both KRAS and NRAS activating mutations, produce characteristic and persistent monocytosis accompanied by high frequency of autoimmunity and antinuclear antibody production [3], symptoms reminiscent of SLE patients.

## Case presentation

**Fig. 1:**
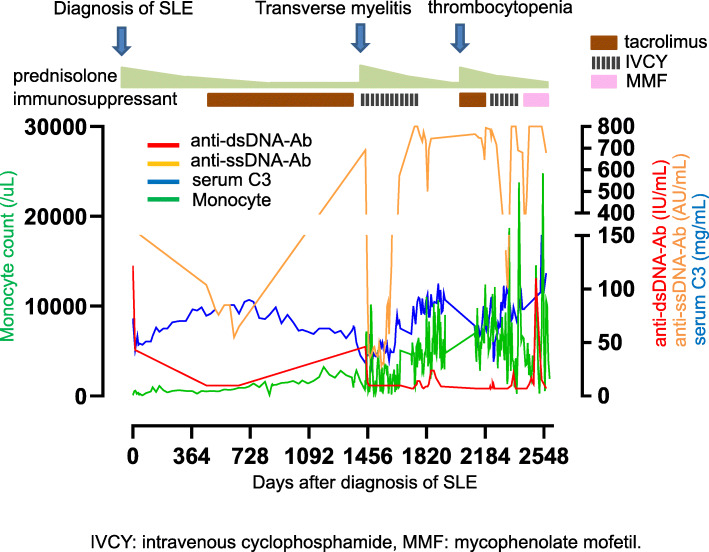
Graph depicts clinical course of the current case. Monocyte count, levels of anti-dsDNA antibody, anti-ssDNA-Ab, and serum C3 of the patient are shown in the graph. Diagnosis and the respective treatment measures are depicted on top of the graph

**Fig. 2:**
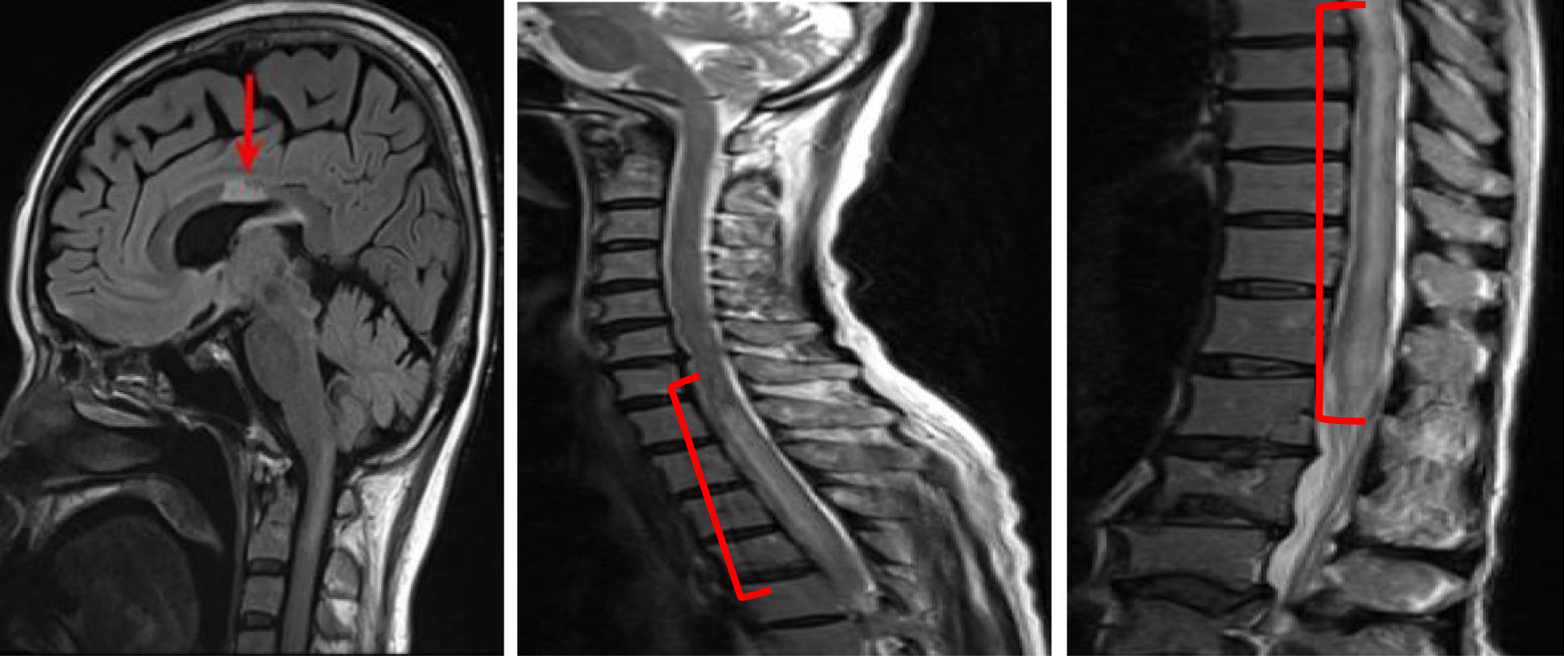
MRI images of the patient’s head and spine. Flair (left panel) and T2-weighed (middle and right panels) images of the patient at the onset of transverse myelitis are shown

**Fig. 3:**
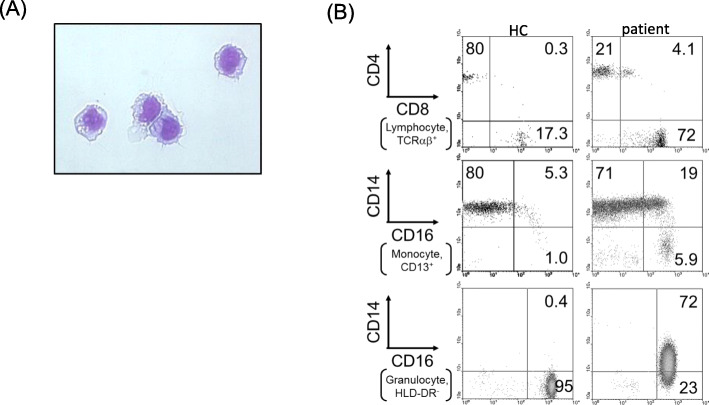
Morphology and phenotypic profiles of blood of the patient. **A** Patient monocytes were sorted by flowcytometry and were Giemsa stained. No abnormalities were found. **B** Flow cytometric analyses of the blood from healthy donor (HC) and the patient. Top panels show the CD4 and CD8 expression profiles of lymphocyte-gated TCRαβ+ cells. CD4+CD8+TCRαβ+ lymphocyte percentage was higher in the patient than in the HC. Middle panels show the CD14 and CD16 expression profiles in the CD13+ cells (monocyte-gated). CD14+CD16+ monocyte percentage was elevated in the patient compared to the HC. Bottom panels show the CD14 and CD16 expression profiles of HLA-DR-negative cells in the large cells defined by Forward and Side scatter plots (granulocyte-gated). CD14+CD16+ granulocyte percentage was augmented in the patient using the HC as reference

**Fig. 4:**
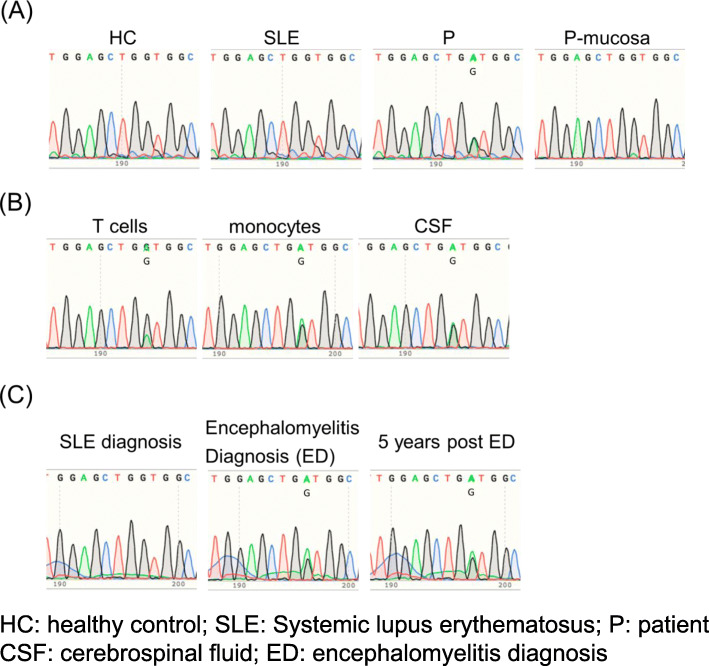
Sanger sequence results of *Kras.* **A T**he chromatograms are showing wild type *Kras* DNA in the PBMC of healthy control (HC), and in another patient with systemic lupus erythematosus (SLE), whereas mutated *Kras* (c.G35A) DNA was detected from PBMC of the patient (P). The same mutation is not detected in mucosa of the patient of interest (P-mucosa). **B** The chromatograms are showing the same mutation site of *Kras* from sorted T cells, monocytes and cerebrospinal fluid (CSF). **C** *Kras* genomic sequence of the patient was wild type when she was diagnosed with SLE. However, *Kras* mutation (c.G35A) manifested spontaneously at the time when she developed encephalomyelitis, and persisted for 5 years after the encephalomyelitis diagnosis (ED)

A 60-year-old female SLE patient was admitted to our hospital due to fever and paralysis of both of her lower legs. Four years before this admission, she had joint swelling on her fingers, wrists, right shoulder, knees, and non-palpable purpura on the forearms and thighs. Thrombocytopenia, elevated erythrocyte sedimentation rate, serum C-reactive protein (CRP), and total serum IgG; as well as reduced complement activity were reported. The patient presented antinuclear antibody (ANA) of 1:320 (homogeneous/speckled type), anti-dsDNA antibody (anti-DNA Ab) of 156 IU/ml, anti-SS-A Ab, circulating immune complex, and prolonged lupus anticoagulant. Based on the 2019 revised European Alliance of Associations for Rheumatology (EULAR) /American College of Rheumatology (ACR) classification criteria, she was diagnosed with SLE. Thrombocytopenia and anti-DNA Ab level were relieved and maintained by the treatment of prednisolone and tacrolimus for more than 4 years (Fig. 1). Four days before the admission, however, she became acutely ill with a fever of 38°. Subsequently, the patient suffered from sudden laxative total paralysis in both lower limbs. Her bladder and rectum showed dysfunction with high intensity signal on the cervical cord of MRI (Fig. 2). Pleocytosis, along with elevated protein, IgG and IL-6 levels in the cerebrospinal fluid (CSF) were detected. The patient presented mild leukocytosis with neutrophil predominance, moderate thrombocytopenia, elevated CRP and serum IgG amount, prolonged active prothrombin time, anti-DNA Ab, anti-ribosomal P antibody and marked reduction of complement activity (Table 1). The diagnosis of transverse myelitis due to the flare of SLE was made; then, standard pulse therapy of methylprednisolone was followed by high-dose of prednisolone, intravenous cyclophosphamide in combination with plasma exchange. In the following months, her refractory neurological and autoimmune symptoms continued to fluctuate, and failed to achieve remission with the appropriate treatments (Fig. 1). The apparent histologically normal monocytes (Fig. 3A) continued to cloud the clinical features of the patient whose monocytosis persisted. Flow cytometry analyses revealed an elevated ratio of CD14+CD16+ atypical monocytes, CD14+CD16+ atypical granulocytes as well as CD4+CD8+TCRαβ+ double positive T cells as compared to the healthy control (Fig. 3B). Increased atypical monocytes excluded chronic myelomonocytic leukemia [4]; and lupus-like symptoms with monocytosis together with these immune-phenotypes reminded us of previously-reported RALD cases, although these immunophenotypes can be seen in SLE cases. Therefore, we performed Sanger sequencing of KRAS and NRAS genes from the patients’ PBMC (Fig. 4A), magnetic-activated cell sorting (MACS)-sorted CD3+ lymphocytes and CD14+ monocytes, together with CSF (Fig. 4B). A point mutation in codon 12 of KRAS (c.G35A, p.G12D) was detected. Since the mutation was almost absent in the buccal mucosa sample of the patient (Fig. 4A), the mutation is likely to be somatic and not germline, as in RASopathies. A backtrack of the patient’s cell-free DNA obtained by the stocked serum samples showed that the mutation manifested at the time of encephalomyelitis diagnosis, and persisted for 5 years (Fig. 4C). This mutation seems to be involved in lupus flare of this case, as it was not detected when she was diagnosed of SLE (Fig. 4C), or in another SLE patient (Fig. 4A).

**Table 1:**
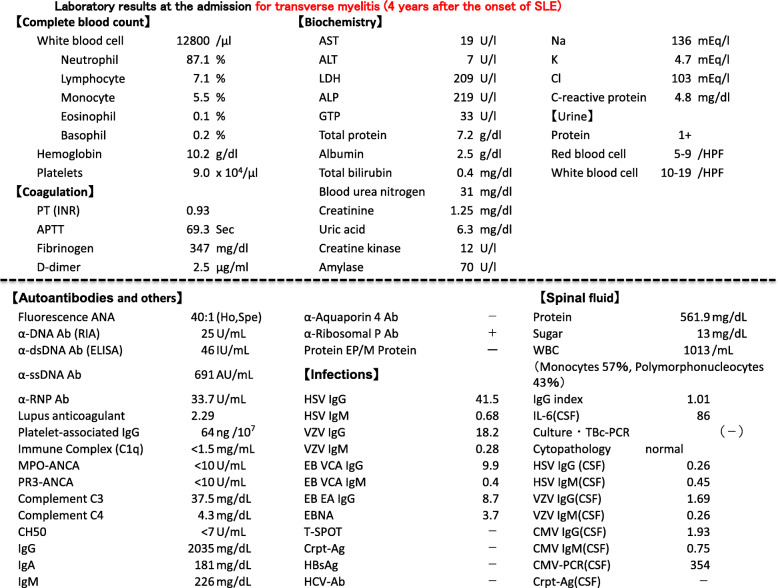
Laboratory results at the admission for transverse myelitis (4 years after the onset of SLE)

## Discussion and conclusions

KRAS G12D mutation is a missense mutation that results in a constitutive activating function [5]. In the immune system, the KRAS molecule regulates normal hematopoiesis [6]. Constitutive active mutation of the RAS protein results in breakdowns of B cell self-tolerance and productions of autoantibody [7], potentially by disruption of the RAS/MAPK regulatory pathway.

Given the many overlapping symptoms between various autoimmune disorders, it is still difficult to have a clear cut in the diagnosis of various similar connective tissue autoimmune diseases. Since RALD patients are known to present symptoms mimicking SLE [8] and KRAS mutation can also give rise to monogenic pediatric SLE [9], one could argue whether this case was SLE or RALD. Although she did not show the typical SLE symptoms like marlar rash or glomerulonephritis, she satisfied the 2019 EULAR/ACR criteria with 22 points (one can be classified as SLE with ≥ 10 points). Therefore, there is no doubt that she had SLE at least at the time of the onset, when we could not detect the mutation using the cell-free DNA in the stocked serum. However, it is not clear whether KRAS mutation was responsible for the recurrence and refractoriness of SLE.

The somatic KRAS mutation detected could be explained by clonal hematopoiesis. Throughout the genome, about 20 somatic mutations are estimated to accumulate in human hematopoietic stem cells (HSCs) each year [10, 11], and about 0.1 mutations in protein-coding exons [12]. By the age of 70, we may harbor from 350,000 to 1,400,000 coding mutations within the HSC pool [13]. When one of these mutations was able to expand due to certain selective advantages, it is coined clonal hematopoiesis. The current accepted definition of clonal hematopoiesis is when a clone reaches a proportion of 4% of cells measured in the peripheral blood [14]. Although most clonal hematopoiesis are linked to hematologic cancers, some mutations found their ways in circulating immune cells like monocytes, which could alter the immune responses of the patients [13]. Unfortunately, we did not collect samples and conduct necessary experiments to check if 4% of her cells in the peripheral blood have this clone, while the patient was residing in our hospital. With that said, when the KRAS mutation was identified, the patient was in her 60s, approaching the age when an acquired mutation would have enough time to accumulate and then expand to reach the classical definition of ~ 4% of cells in the peripheral blood, or of a variant allele frequency of over 2% in the blood [14]. Comparing to genes that are more commonly mutated in clonal hematopoiesis (DNMT3A, 48.3%), KRAS mutations contribute to about 1.3% of the current cases discovered [9].

Unlike the limited number of genes such as DNMT3A, which enhances self-renewal of stem and progenitor cells [15]; KRAS, a small GTPase could be causing some of the resistance in the treatments for our patient. Autoreactive immature B cells usually reside in the bone marrow, but Teodorovic et al. have shown that activation of Ras can change this pattern, altering the Ras-erk pathway, and resulting in secretion of autoantibodies. Although not the same mutation (N-RasD12), KRAS (G12D) is also an activating mutation, which could give rise to the production of autoantibodies. Using serial transplantation mouse studies might be able to help elucidate KRAS (G12D) function in the treatment resistance. Given the molecular complexity of different autoimmune diseases, we could only deduce that this is a case of refractory SLE with monocytosis, accompanying a somatic KRAS mutation. Since other genetic alterations can also trigger SLE-like and RALD-like conditions, we would need to conduct a more in-depth investigation of other potential somatic mutations such as PTPN11, RAF, SHOC2 and SOS1 to understand this case better. Even so, we believe this case provides an additional reference for understanding complicated SLE pathogenesis.

## Data Availability

No datasets were generated or analyzed during the current study.

## Abbreviations

SLE: Systemic lupus erythematosus
ACR: American College of Rheumatology
CSF: Cerebrospinal fluid
KRAS: Kirsten Rat Sarcoma Proto-Oncogene
GTPase; NRAS: Neuroblastoma rat sarcoma proto-oncogene
GTPase; MAPK: Mitogen-activated protein kinase
PTPN11: Protein tyrosine phosphatase non-receptor type 11
RAF: Raf-1 proto-oncogene, serine/threonine kinase
SHOC2: Suppressor of clear homolog (C. Elegans)
LRRSP2: Leucine-rich repeat scaffold protein 2
SOS1: Son of Sevenless Homolog
Ras/Rac: Guanine nucleotide exchange factor 1
RALD: Ras-associated autoimmune leukoproliferative disorder
ALPS: Autoimmune lymphoproliferative syndrome
FAS: Fas cell surface death receptor
CRP: C-reactive protein
IgG: Immunoglobulin G
ANA: Antinuclear antibody
Anti-DNA Ab: Anti-DNA antibody
MACS: Magnetic-activated cell sorting
PBMC: Peripheral blood mononuclear cells
